# Study-level factors associated with hematoma after ultrasound-guided vacuum-assisted breast lesion excision: a systematic review and meta-analysis using a T-P-B framework

**DOI:** 10.3389/fonc.2026.1828439

**Published:** 2026-06-19

**Authors:** Yunzhi Shen, Xinran Shao, Shuai Ma, Yihan Sun, Jinrui Liu, Pingdong Sun, Yuxia Jiang, Xiang Fei, Ying Zhang, Yang Gao, Dongning Bi, Jianchun Cui, Xingai Ju, Dongxiao Zhang

**Affiliations:** 1 Dalian Medical University, Dalian, China; 2 Department of Thyroid and Breast Surgery,People’s Hospital of Liaoning Province, Shenyang, China; 3 Graduate Training Base of Liaoning Provincial People’s Hospital, China Medical University, Shenyang, China; 4 Department of Cardiology, People’s Hospital of Liaoning Province (People’s Hospital of China Medical University), Shenyang, China; 5 Department of General Surgery, Liaoyang County Central Hospital, Liaoyang, China; 6 Graduate Training Base of Liaoning Provincial People’s Hospital, Jinzhou Medical University, Shenyang, China; 7 Department of General Medicine, People’s Hospital of Liaoning Province (People’s Hospital of China Medical University), Shenyang, China; 8 Department of Breast Surgery, Beijing Hospital of Traditional Chinese Medicine, Capital Medical University, Beijing, China

**Keywords:** hematoma, meta-analysis, risk factors, systematic review, vacuum-assisted breast biopsy, vacuum-assisted breast excision

## Abstract

**Objective:**

To systematically summarize study-level factors associated with postoperative hematoma after ultrasound-guided vacuum-assisted breast lesion excision(VAE) using a vacuum-assisted breast biopsy (VABB) system, and to organize the available evidence according to a novel T-P-B framework comprising tumor-related, position-related, and breast- or peri-procedural management-related factors.

**Methods:**

A systematic search was conducted in PubMed, China National Knowledge Infrastructure, Wan fang Data, VIP Database, and Elsevier Clinical Key for studies published between January 1995 and October 2025. Observational studies investigating risk factors for hematoma after VAE using a VABB system were included. Study-derived exposure categories and cutoff values were retained. Study quality was assessed using the Newcastle–Ottawa Scale (NOS). Meta-analysis was performed using R (version 4.5.1). Effect sizes were expressed as odds ratios (ORs) with corresponding 95% confidence intervals (CIs) and were pooled using the inverse-variance method. Heterogeneity was assessed using the I² statistic, and a random-effects model was applied. Sensitivity analysis was conducted by sequentially excluding individual studies (leave-one-out analysis). A two-sided *p* value < 0.05 was considered statistically significant.

**Results:**

A total of 11 retrospective studies involving 3,516 patients who underwent VAE using a VABB system were included, among whom 444 cases of postoperative hematoma were reported. Five study-level factors were identified. Meta-analysis demonstrated that, within the T dimension, tumor numbers (OR = 4.21, 95% CI = 2.59–6.85), numbers of cutting passes(OR=3.87,95%CI=2.16-6.95)were significantly associated with hematoma formation. Within the P dimension, non-moderate tumor depth, including superficial or deep tumor, was associated with postoperative hematoma (OR = 4.39, 95% CI: 1.21–15.92), as was higher vascularity grade (OR = 2.60, 95% CI: 1.42–4.76). Within the B dimension, postoperative compression duration <48 h was associated with hematoma formation (OR = 4.34, 95% CI: 2.53–7.45). Sensitivity analyses showed generally consistent directions of association.

**Conclusions:**

This systematic review and meta-analysis identified five study-level factors associated with postoperative hematoma after VAE using a VABB system: tumor numbers, a higher number of cutting passes, non-moderate tumor depth including superficial or deep tumor, higher vascularity grade, and postoperative compression duration <48 h. The T-P-B framework may provide a clinically interpretable structure for organizing these factors and informing perioperative risk assessment.

## 1 Introduction

In 1995, Fred Burbank and Mark Retchard developed the vacuum-assisted breast biopsy (VABB) system, which was introduced into China in 1997 (1). In 2002, the U.S. Food and Drug Administration approved the use of VABB for the excision of selected benign breast lesions, thereby expanding its clinical application from diagnostic tissue sampling to therapeutic lesion removal (2). In recent years, with the publication of domestic guidelines and expert consensus statements, ultrasound-guided vacuum-assisted breast lesion excision, also referred to as vacuum-assisted excision (VAE) using a vacuum-assisted breast biopsy system, has been increasingly used in clinical practice in China.

Although VAE is generally considered minimally invasive and cosmetically favorable, procedure-related complications remain clinically relevant. Bleeding and postoperative hematoma are among the most common complications (3). A meta-analysis reported that the incidence of postoperative hematoma after VABB/VAE was approximately 9% (4). Most postoperative hematomas are mild or moderate and can be managed conservatively with compression or observation. However, rare but severe vascular complications resulting in large hematoma formation have also been reported after vacuum-assisted breast procedures (5), indicating that hematoma-related complications still warrant clinical attention. Existing evidence is mainly derived from retrospective observational studies, and the reported risk factors are scattered across different clinical dimensions. As a result, the current evidence remains fragmented, and no systematic framework has been established to integrate these factors for perioperative risk assessment.

To address this gap, the present systematic review and meta-analysis aimed to summarize study-level factors associated with postoperative hematoma after VAE using a VABB system. From the perspective of clinical practice, we proposed a novel T-P-B framework to organize the available evidence. In this framework, “T” refers to tumor-related factors reflecting tumor burden and excision demand; “P” refers to position-related factors describing the anatomical relationship between the tumor and surrounding structures; and “B” refers to breast- and peri-procedural management-related factors. This framework was not derived from a previously validated prediction model; rather, it was developed as an organizational structure to integrate available evidence and to facilitate perioperative risk assessment.

## 2 Materials and methods

### 2.1 Literature search and sources

This study was conducted in accordance with the PRISMA Statement. The protocol of this systematic review and meta-analysis was not prospectively registered in PROSPERO or other public registries. To address the issue of methodological transparency, we reported the literature search, eligibility criteria, study selection, data extraction, quality assessment, and statistical analysis procedures in detail according to the PRISMA 2020 statement. The PRISMA 2020 checklist was provided as a **Supplementary Document 2**. A systematic literature search was performed in PubMed, China National Knowledge Infrastructure (CNKI), Wan fang Data, VIP Database, and Elsevier Clinical Key. The search covered publications from January 1, 1995 to October 25, 2025. The search period began in 1995 because vacuum-assisted breast biopsy was first introduced in the mid-1990s. However, all studies that finally met the eligibility criteria and were included in the quantitative synthesis were published between 2016 and 2025.

Both Medical Subject Headings (MeSH) and free-text terms were used in combination. The search fields and Boolean operators were adjusted according to the characteristics of each database. Additionally, the reference lists of included studies and relevant review articles were manually screened to identify potentially eligible studies. An example of the search strategy was:(“Vacuum-Assisted Biopsy”[MeSH] OR Mammotome OR “vacuum assisted breast biopsy” OR VABB) AND (“Hematoma”[MeSH] OR hemorrhage OR bleeding) AND (“Risk factors”[MeSH] OR “risk factor” OR “predictor” OR “prediction”). The search strategy was primarily based on device- and technique-related terms because the same vacuum-assisted biopsy system is used for both diagnostic biopsy and therapeutic lesion removal or excision. The complete search strategies for each database are provided in **Supplementary Table 1**.

### 2.2 Inclusion and exclusion criteria

#### 2.2.1 Inclusion criteria

1. The study population consisted of patients undergoing ultrasound-guided vacuum-assisted breast lesion removal or excision using a vacuum-assisted biopsy system, including procedures described in the original studies as VABB, VAE, Mammotome excision, EnCor minimally invasive excision, or minimally invasive rotary excision.
2. The study design was observational, including cohort studies (prospective or retrospective) or case–control studies;
3. The study clearly reported postoperative bleeding or hematoma as an outcome and investigated at least one associated risk factor, such as tumor number, tumor depth or location, vascularity grade, tumor size, or breast morphology;
4. The study provided sufficient data for effect size synthesis, including odds ratios (ORs), risk ratios (RRs), or hazard ratios (HRs) with corresponding 95% confidence intervals (CIs), or reported original data that allowed calculation of effect estimates (e.g. two-by-two contingency table data).

#### 2.2.2 Exclusion criteria

1. Reviews, commentaries, case reports, conference abstracts, duplicate publications, or studies with obviously overlapping data and incomplete information (when data overlap was identified, the study with the larger sample size and more complete data was preferentially included);
2. Animal experiments or *in vitro* studies;
3. Articles published in languages other than Chinese or English;
4. Studies that did not report postoperative bleeding or hematoma outcomes, or from which the effect estimates required for meta-analysis could not be extracted or calculated.
5. Studies involving conventional diagnostic core needle biopsy alone, open surgical excision, or procedures in which outcomes of vacuum-assisted excision could not be separated from other techniques were excluded.

### 2.3 Quality assessment and data extraction

Study selection was conducted independently by two reviewers in two stages. First, titles and abstracts were screened to exclude clearly irrelevant records. Second, the full texts of potentially eligible articles were assessed according to the predefined inclusion and exclusion criteria. Data extraction and methodological quality assessment were also performed independently by the same two reviewers. Disagreements at any stage were resolved through discussion; when consensus could not be reached, a third reviewer was consulted for adjudication. Inter-reviewer agreement was not formally quantified using Cohen’s kappa statistic. The methodological quality of the included studies was assessed using the Newcastle–Ottawa Scale (NOS), which evaluates studies from three domains: selection of study participants, comparability between groups, and assessment of outcomes or exposures. The NOS score ranges from 0 to 9 points, with scores of 0–3 indicating low quality, 4–6 indicating moderate quality, and 7–9 indicating high quality. The following data were extracted from each eligible study:

1. Basic study characteristics: first author, year of publication, study design, and source of the study population;
2. Study population and outcomes: total sample size, number of bleeding or hematoma cases, and the definition and assessment time window for bleeding or hematoma;
3. Exposure variables and definitions: such as tumor number, tumor size, tumor depth or location, vascularity grade and other available factors. The dichotomization thresholds were extracted from the original studies rather than defined by the authors of the present meta-analysis.
4. Effect estimates: adjusted odds ratios (aORs) with corresponding 95% confidence intervals (CIs) were preferentially extracted. If only univariate ORs or raw data were reported, the unadjusted ORs were extracted, or crude ORs were calculated from the original data using the meta package in R. These estimates were subsequently evaluated through sensitivity analyses. Because adjusted and crude ORs differ in their degree of confounding control, pooled estimates combining these measures were interpreted cautiously as associations rather than independent causal effects.

In addition, to further evaluate potential procedural confounders and sources of clinical heterogeneity, we also extracted procedural information when available, including the procedural terminology used in the original studies, device type, needle gauge, anesthesia method, puncture approach, and operator experience. However, most studies did not provide sufficient effect estimates or comparable analytical data regarding their associations with postoperative hematoma. Consequently, these procedural factors could not be included in the quantitative synthesis and are presented descriptively in **Supplementary Table 2**.

### 2.4 Statistical analysis

Statistical analyses were performed using R software (version 4.5.1). For dichotomous outcomes, effect sizes were expressed as odds ratios (ORs) with corresponding 95% confidence intervals (CIs). Reported ORs were logarithmically transformed, and ln(OR) values were used as input parameters for the meta-analysis. Standard errors (SEs) were calculated from the corresponding 95% CIs. When both adjusted and crude estimates were available, adjusted estimates were used in the primary analysis. For studies that reported only raw data, crude ORs were calculated and included to preserve the available evidence. Sensitivity analyses excluding crude ORs were considered when a sufficient number of adjusted estimates remained for a given risk factor. When this was not feasible because of the small number of studies, the potential influence of combining adjusted and crude estimates was addressed qualitatively. If different types of effect estimate for the same risk factor were reported across studies, ORs were preferentially pooled whenever possible. Heterogeneity among studies was assessed using Cochran’s Q test and the I² statistic. Considering that most included studies were retrospective observational studies and that clinical and methodological heterogeneity was expected, a random-effects model using restricted maximum likelihood (REML) estimation was preferentially applied for pooled analyses. When substantial heterogeneity was observed and could not be reasonably explained by clinical considerations or subgroup analyses, a descriptive synthesis was performed instead of quantitative pooling. Sensitivity analyses were conducted using a leave-one-out approach to evaluate the influence of each individual study on the overall effect estimate. Because fewer than ten studies were included for each risk factor, publication bias was not quantitatively assessed using Egger’s test or funnel plot analysis. Instead, potential publication bias was qualitatively discussed in the Discussion section. Statistical significance was defined as a two-sided *P* value < 0.05. Notably, the reference and exposure categories used in each pooled analysis followed the definitions reported in the original studies. No new thresholds were generated during the meta-analysis.

## 3 Results

### 3.1 Study selection

A total of 744 records were initially identified through the database search. After removing 78 duplicate records, 666 records remained for title and abstract screening. Following this step, 636 studies were excluded. The full texts of the remaining 30 articles were assessed for eligibility, of which 19 were excluded due to the absence of outcome data or lack of analysis of factors associated with postoperative hematoma. Ultimately, 11 studies of VAE using a VABB system were included in the quantitative synthesis. Although the terminology varied among the original studies, all included studies used ultrasound-guided vacuum-assisted biopsy systems for percutaneous lesion removal or excision. Studies involving conventional diagnostic biopsy alone were not included.

**Figure 1:**
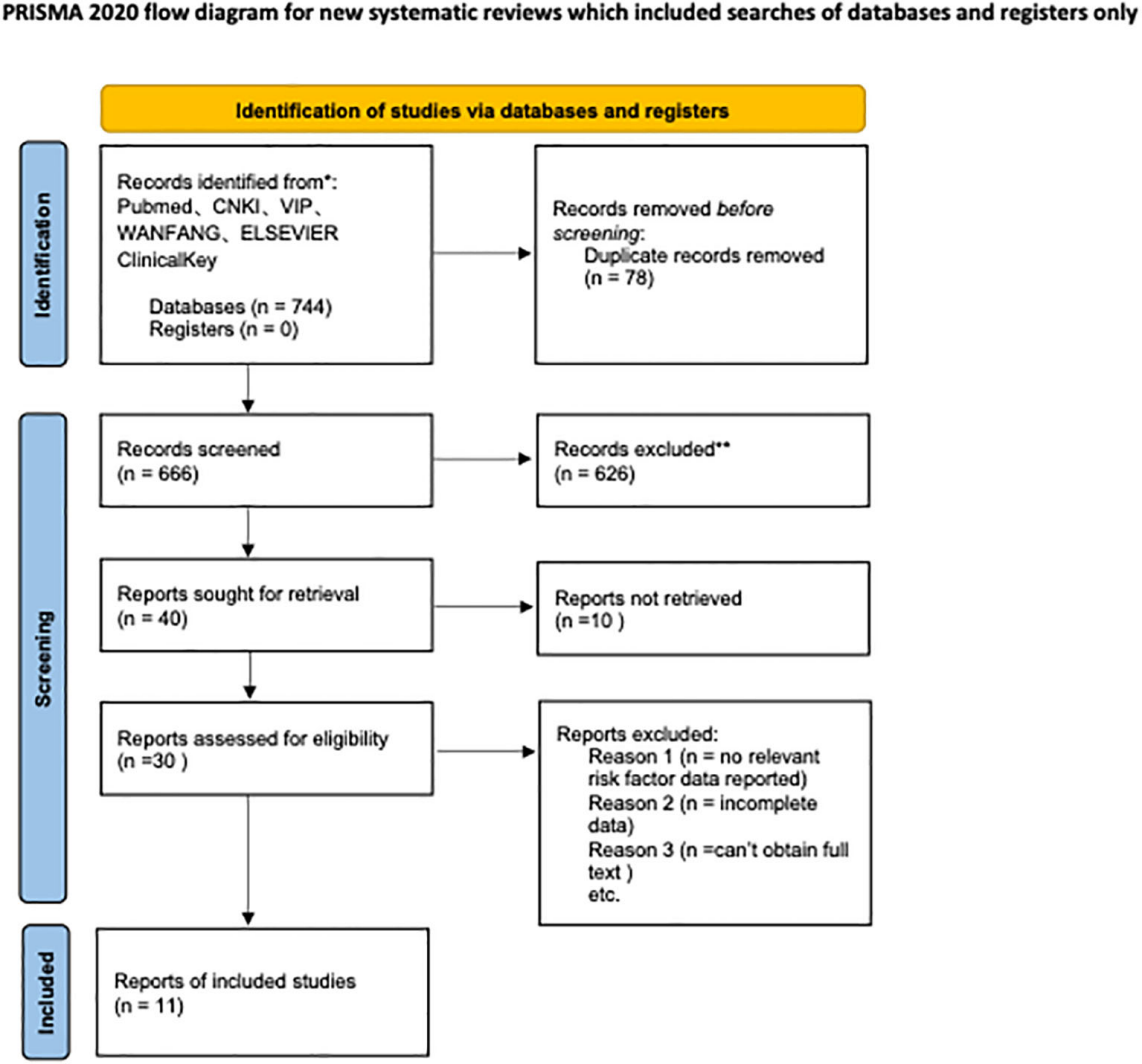
PRISMA flow diagram of the literature selection process.

The study selection process followed the PRISMA 2020 flow diagram (6), as shown in **Figure 1**.

### 3.2 Study characteristics and quality assessment

The basic characteristics of the included studies are summarized in **Table 1**, and the results of the quality assessment are presented in **Supplementary Table 3**. A total of 11 retrospective cohort studies were included, published between March 2016 and April 2025, involving 3,516 patients who underwent VAE using a VABB system.

**Table 1:** Characteristics of the included studies.

First author	Year	Study design	Sample size (n)	Hematoma cases (n)	Evaluated risk factors	Source of effect estimates	Definition of hematoma
Dong Yunyun (7)	2025	Retrospective cohort	137	Not available	3	aOR, 95%CI	Ultrasound-detected fluid collection >1 cm at the surgical site within 24–48 h postoperatively
Zhang Ding (8)	2024	Retrospective cohort	112	70	3,4,5	aOR, 95%CI	Ultrasound-detected hypoechoic area >1 cm at the surgical site at 24 h postoperatively
Cui Guangjun (9)	2023	Retrospective cohort	132	95	2	aOR, 95%CI	Ultrasound-detected hypoechoic area >1 cm at the surgical site at 24 h postoperatively
Wang Pei (10)	2022	Retrospective cohort	80	6	1,3,4,5	aOR, 95%CI	Ultrasound-detected hypoechoic area >1 cm at the surgical site at postoperative day 3
Dong Yunyun (11)	2021	Retrospective cohort	68	44	2,3	aOR, 95%CI	Ultrasound-detected hypoechoic area >1 cm at the surgical site at 24 h postoperatively
Zheng Jianwei (12)	2020	Retrospective cohort	293	39	1,4	aOR, 95%CI	Ultrasound-detected hypoechoic area >1 cm at the surgical site at 24 h postoperatively
Li Min (13)	2020	Retrospective cohort	190	15	4,5	aOR, 95%CI	Ultrasound-detected anechoic area with a long-axis diameter >1 cm at the surgical site
Lv Hao (14)	2019	Retrospective cohort	585	32	1	crudeOR	Ultrasound-confirmed hematoma >10 mL at the surgical site at 24 h postoperatively
Yao Chun (15)	2017	Retrospective cohort	412	43	1,3,4,5	aOR, 95%CI	Ultrasound-detected anechoic area with a long-axis diameter >1 cm at the surgical site
Liu Shu (16)	2017	Retrospective cohort	1267	70	1	crudeOR	Not Available
Huo Huiping (17)	2016	Retrospective cohort	232	30	1	aOR, 95%CI	Ultrasound evaluation of hematoma progression within 24–48 h postoperatively

Coding of risk factors: 1 = tumor number; 2 = non-moderate tumor depth (distance from the tumor center to the skin); 3 = vascularity grade; 4 = duration of breast compression; 5 = number of cutting passes.

Dichotomization thresholds used in the original studies: 1 = tumor number: 1 vs. >1; 2 = non-moderate tumor depth (distance from the tumor center to the skin): 0.65–1.5 cm vs. ≤0.65 cm or ≥1.5 cm; 3 = vascularity grade: grade 0–1 vs. grade 2–3; 4 = duration of breast compression: ≥48 h vs. <48 h; 5 = number of cutting passes: <22 vs. ≥22.

Effect size definitions: adjusted odds ratio (aOR), defined as the multivariable-adjusted odds ratio reported in the original study; crude odds ratio (crude OR), defined as the unadjusted odds ratio calculated in the present study based on the raw data, namely the 2 × 2 contingency table provided in the original article, using the meta package in R.

The study by Dong Yunyun et al. (2025) did not report the number of patients with postoperative hematoma. Instead, hematoma outcomes were described according to the number of nodules. Because the remaining studies reported hematoma events at the patient level, the number of hematoma cases from this study was not included in the total hematoma event count. Therefore, the total of 444 postoperative hematoma cases was calculated from the remaining 10 studies with available patient-level hematoma counts. The definition of “hematoma” and the assessment time window varied across studies. In most studies, hematoma was defined as a hypoechoic or fluid-containing area >1 cm in the surgical region detected by ultrasound within 24–48 hours postoperatively. A few studies defined hematoma based on a hematoma volume >10 mL or did not clearly specify the diagnostic criteria. These variations may have affected the consistency of outcome assessment and could represent a potential source of heterogeneity.

The Newcastle–Ottawa Scale (NOS) assessment showed that the included studies had total scores ranging from 5 to 9, indicating overall moderate to high methodological quality (see **Supplementary Table 3**).Some studies received lower scores in the domain of “adequacy or duration of follow-up,” which may be related to the fact that postoperative hematoma following VAE using a VABB system typically occurs within a short postoperative period, as well as incomplete follow-up records or inconsistent follow-up time points across studies.

### 3.3 Results of the meta-analysis

**Figure 2:**
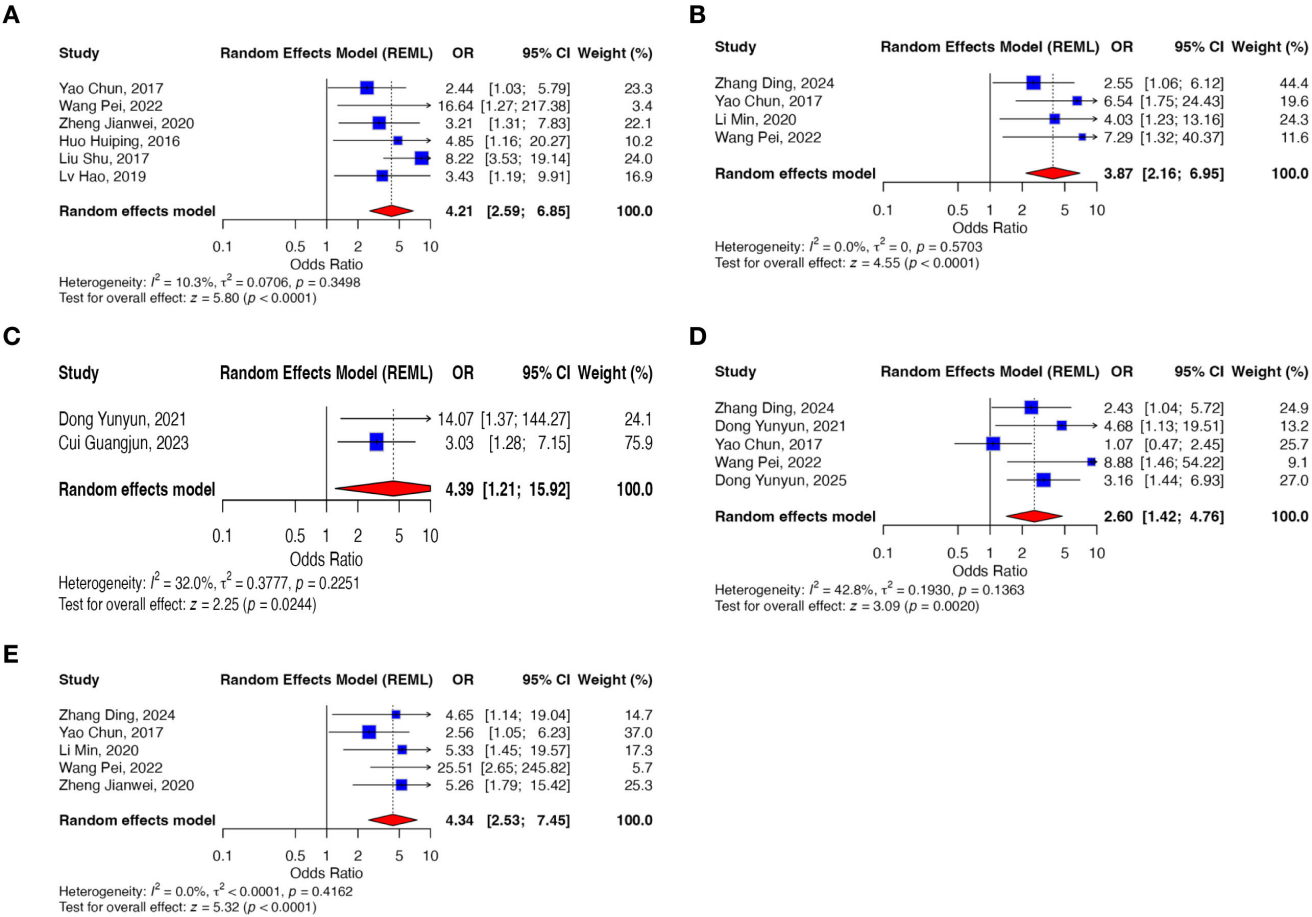
Forest plots illustrating the associations between study-level factors and postoperative hematoma after VAE using a VABB system: **(A)** tumor number, **(B)** number of cutting passes, **(C)** non-moderate tumor depth (distance from tumor center to skin), **(D)** vascularity grade and **(E)** duration of breast compression. **(A)** Forest plot of tumor number. **(B)** Forest plot of number of cutting passes. **(C)** Forest plot of non-moderate tumor depth (distance from tumor center to skin). **(D)** Forest plot of vascularity grade. **(E)** Forest plot of duration of breast compression.

Meta-analysis was performed using R (version 4.5.1). The pooled effect sizes for each factor and the corresponding forest plots are shown in **Figures 2A–E**, ordered by tumor number, number of cutting passes, non-moderate tumor depth, vascularity grade, and duration of breast compression.

#### 3.3.1 T dimension

For tumor number (**Figure 2A**), heterogeneity testing indicated low heterogeneity among the included studies (I² = 10.3%, P = 0.3498). Under the random-effects model, multiple tumors were associated with a higher likelihood of postoperative hematoma compared with a single tumor (OR = 4.21, 95% CI: 2.59–6.85, Z = 5.80, p < 0.0001). Notably, two studies in this model contributed crude ORs because adjusted estimates were unavailable. For the number of cutting passes (**Figure 2B**), no substantial between-study heterogeneity was observed (I² = 0.0%, p = 0.5703). A higher number of cutting passes was also associated with postoperative hematoma (OR = 3.87, 95% CI: 2.16–6.95, Z = 4.55, p < 0.0001).

#### 3.3.2 P dimension

For non-moderate tumor depth (distance from the tumor center to the skin) (**Figure 2C**), specifically including superficial or deep tumor, the pooled analysis showed an association between non-moderate tumor depth and postoperative hematoma (OR = 4.39, 95% CI: 1.21–15.92, Z = 2.25, p = 0.0244), with low-to-moderate heterogeneity (I² = 32.0%, p = 0.2251). For vascularity grade (**Figure 2D**), moderate between-study heterogeneity was observed (I² = 42.8%, p = 0.1363). Higher vascularity grade was associated with a higher likelihood of postoperative hematoma (OR = 2.60, 95% CI: 1.42–4.76, Z = 3.09, p = 0.0020).

#### 3.3.3 B dimension

Duration of postoperative compression was analyzed (**Figure 2E**). No substantial between-study heterogeneity was observed (I² = 0.0%, p = 0.4162). Compared with compression duration ≥48 h, compression duration <48 h was associated with a higher likelihood of postoperative hematoma (OR = 4.34, 95% CI: 2.53–7.45, Z = 5.32, p < 0.0001).

### 3.4 Sensitivity analysis and publication bias

To assess the robustness and reliability of the meta-analysis results, a leave-one-out sensitivity analysis was performed for each risk factor using R software (version 4.5.1).

The results showed that for the models of tumor number, number of cutting passes, tumor depth, and vascularity grade and duration of breast compression. The direction of the pooled odds ratios (ORs) remained consistent after sequentially excluding each individual study. The magnitude of the pooled effect estimates showed only limited variation, indicating that the main findings were not driven by any single study and demonstrated acceptable stability (see **Supplementary Figures A–E**). For the vascularity grade model, exclusion of the study by Yao et al. reduced the heterogeneity to I² = 0% and increased the pooled effect estimate to OR = 3.29 (95% CI: 1.97–5.50). This finding suggests that the study contributed substantially to between-study heterogeneity. However, after sequential exclusion, the pooled association remained statistically significant, supporting the overall observed association. In addition, Dong Yunyun et al. (2025) did not report the number of hematoma cases at the patient level but contributed an adjusted effect estimate to the vascularity grade model, we specifically evaluated its influence in the leave-one-out sensitivity analysis. After excluding it, the association between higher vascularity grade and postoperative hematoma remained directionally consistent and statistically significant (OR = 2.57, 95% CI: 1.11–5.91, p = 0.0270). Because crude ORs were used only in the analysis of tumor number, an additional sensitivity analysis was performed after excluding the two studies that contributed crude ORs. After exclusion, four studies remained in the adjusted-estimate-only analysis. The pooled association between tumor number and postoperative hematoma remained statistically significant, with an OR of 3.29 (95% CI: 1.89–5.74, p < 0.0001), and no between-study heterogeneity was observed (I² = 0.0%, p = 0.5178) (**Supplementary Figure F**).

Because fewer than 10 studies were included for each risk factor, funnel plots and Egger’s regression tests were not performed to assess publication bias, given their limited statistical power and potential instability under such conditions, and the statistical power of the pooled analyses was limited. Therefore, the present findings should be interpreted as exploratory evidence of association.

## 4 Discussion

In this systematic review and meta-analysis, we summarized available observational evidence on factors associated with postoperative hematoma after VAE using a VABB system. The pooled results suggested that hematoma occurrence was associated with factors from different clinical dimensions, including tumor burden, anatomical location, and postoperative management. Rather than interpreting these variables as isolated predictors, we organized them using the T-P-B framework to improve clinical interpretability. Its purpose is to link study-level evidence with perioperative decision-making, including preoperative risk recognition, intraoperative procedural planning, and postoperative hematoma prevention.

### 4.1 Study-level factors of T-P-B framework

#### 4.1.1 T dimension

Tumor numbers was associated with an increased incidence of postoperative hematoma (OR = 4.21, 95% CI: 2.59–6.85). During procedures involving multiple lesions, surgeons often need to establish multiple puncture tracts or perform repeated rotational excisions through a single tract. This may increase the extent of regional breast parenchymal injury and consequently raise the total number of transected vessels. As a result, it becomes more difficult to apply uniform and effective compression to multiple residual cavities after the procedure, leading to insufficient hemostatic pressure and a higher likelihood of postoperative hematoma formation. In the tumor number analysis, two studies contributed crude ORs, after excluding these studies, the association between tumor number and postoperative hematoma remained statistically significant and directionally consistent, although the pooled effect estimate was attenuated. Notably, one of the excluded studies also did not provide a clear definition of postoperative hematoma; therefore, this analysis also partially addressed the potential influence of unclear outcome definition in the tumor-number model.

A higher number of cutting passes was associated with an increased risk of postoperative hematoma (OR = 3.87, 95% CI: 2.16–6.95). Each cutting and sampling pass involves penetration and rotational excision through the breast tissue; therefore, as the number of passes increases, the probability of tissue or vascular transection during the procedure also rises, which may lead to intraoperative bleeding and subsequently predispose patients to postoperative hematoma formation. This variable may partly reflect larger lesion volume, multiple or irregularly shaped lesions, a larger residual cavity, or the need for repeated tissue removal. Previous study has reported that larger lesion volume is associated with a higher risk of clinically significant hematoma (18). Larger lesions tend to result in a greater residual cavity after excision, which weakens the recoil and compressive effect of the surrounding breast parenchyma (19). Consequently, minor bleeding may persist and gradually accumulate, eventually leading to hematoma formation. In our review, tumor size was not quantitatively synthesized because the original studies used different dichotomization thresholds and reporting formats (such as 23.4mm (12),4.1cm^3^ (13)) which prevented reliable harmonization of exposure categories. For this reason, we did not include tumor size as a pooled factor. Nevertheless, the association between a higher number of cutting passes and postoperative hematoma may indirectly highlight the clinical importance of lesion burden and excision demand.

#### 4.1.2 P dimension

Tumor depth was associated with postoperative hematoma (OR = 4.39, 95% CI: 1.21–15.92). Deeply located tumors, particularly those adjacent to the pectoral muscle, increase the risk of muscle injury during needle insertion, which may result in intraoperative bleeding. Conversely, superficially located tumors are close to the subcutaneous vascular network, and excessive manipulation in this region may damage small vessels, thereby increasing the risk of hematoma formation. These mechanisms are consistent with recent evidence from Tan et al., who reported that skin-to-lesion distance <5 mm was associated with postoperative complications after ultrasound-guided VAE. Although their outcome included overall postoperative complications rather than hematoma alone, hematoma was the most frequent complication in that cohort, supporting the clinical relevance of superficial lesion location (20). In a machine learning–based predictive model for VABB complications, a tumor-to-skin or tumor-to-pectoralis muscle distance also has been identified as an important predictor of ecchymosis and hematoma (21), supporting the clinical relevance of spatial proximity as a risk factor. Several original studies have also suggested that lesion depth or skin-to-lesion distance may be associated with hematoma (7); however, the cutoff values used to define superficial or deep lesions varied across studies(such as ≥1.5cm or <0.7cm). Because these exposure definitions were not sufficiently consistent, lesion depth could not be further harmonized beyond the study-defined categories used in the pooled analysis. This limitation should be considered when interpreting the pooled estimate.

At the vascularity level (OR = 2.60, 95% CI: 1.42–4.76), higher perilesional vascularity grades (Adler grade II–III) were positively associated with the occurrence of postoperative hematoma following VAE. Tumors with abundant vascular structures within the surrounding tissues, or those located near blood vessels, may increase the likelihood of vascular transection during the vacuum-assisted excision procedure, thereby predisposing patients to persistent bleeding or hematoma formation. In addition, rich intralesional vascularity may indicate a higher probability of malignancy (22). Therefore, the biological nature of the tumor should be carefully reassessed to determine whether VABB remains an appropriate treatment option. The influence of Dong Yunyun et al. (2025), which lacked patient-level hematoma counts, was evaluated in the sensitivity analysis and did not materially alter the direction of the association. Previous study has suggested that lesions located near peripheral or central vascular structures may be associated with a higher likelihood of bleeding-related complications, including hematoma (23). This indicates that the incidence of hematoma may differ among different breast quadrants.

#### 4.1.3 B dimension

Insufficient duration of compression bandaging (<48 h) was associated with a higher likelihood of postoperative hematoma formation. When compression is maintained for only a short period, the residual cavity created after excision cannot receive adequate and sustained pressure. Premature removal of the bandage and early upper-limb activity may disrupt immature thrombi before they become stabilized, thereby predisposing patients to recurrent bleeding or persistent oozing. Conversely, this finding suggests that maintaining adequate postoperative compression (≥48 h) represents a simple, effective, and cost-efficient protective measure.

In addition, a study has indicated that large and lax breast morphology may predispose patients to hematoma formation (12). In breasts with loose tissue, effective postoperative compression can be difficult to maintain, and intramammary lesions may be harder to stabilize during the procedure, leading to relative displacement. These factors may collectively increase the risk of postoperative hematoma. Although some studies have reported inconsistent findings (17), breast morphology remains a potential factor.

### 4.2 Clinical interpretation of the T-P-B framework

The T-P-B framework proposed in this study was developed from a clinical practice perspective to organize heterogeneous hematoma-associated factors into three perioperative dimensions. This framework may help clinicians perform structured preoperative and perioperative risk assessment. More importantly, it allows clinicians to distinguish between intrinsic or less modifiable risk-related factors and modifiable peri-procedural factors. Such distinction may help guide targeted preventive strategies and may contribute to improved clinical management.

Within the T dimension, tumor number represents an intrinsic lesion-related factor and may reflect a greater excision burden. To minimize this risk, adequate compression should be applied after removal of the first lesion to ensure the absence of active bleeding before proceeding with subsequent excisions. In addition, surgical management may follow the principle of operating according to increasing Breast Imaging Reporting and Data System (BI-RADS) categories, from lower to higher levels, as recommended in the 2021 clinical guidelines (24).

In contrast, the number of cutting passes is more closely related to procedural management. Although the number of cutting passes may partly reflect tumor burden, lesion size, or excision demand, it is also influenced by operator planning, needle positioning, and excision strategy. Therefore, operators should carefully assess the spatial relationship between the cutting groove of the vacuum-assisted device and the lesion to achieve precise excision (25).

Within the P dimension, non-moderate tumor depth, including superficial or deep lesion locations, and higher vascularity grade are mainly intrinsic anatomical factors. These factors cannot be directly modified before the procedure, but they may alert operators to adopt more cautious and individualized procedural strategies. During operation, hydrodissection or tumescent local anesthesia, in which fluid or anesthetic solution is injected to increase the distance between the lesion and the skin, pectoralis muscle, or adjacent vessels, may help create a safer operative plane and reduce intraoperative bleeding (26). Previous procedural recommendations have also emphasized careful puncture-route planning and avoidance of vascular structures during vacuum-assisted breast procedures (27).

Within the B dimension, postoperative compression duration is a more directly modifiable factor. Standardized and adequate compression may represent a simple and low-cost strategy to reduce hematoma formation, particularly in patients with intrinsic high-risk features, such as multiple lesions, non-moderate tumor depth, or higher vascularity grade. In addition to external compression, one study reported that space-occupying compression of the excision cavity using a Foley catheter could effectively reduce the incidence of hematoma (28, 29). These findings suggest that postoperative management can be individualized according to preoperative and intraoperative risk profiles.

### 4.3 Methodological and clinical sources of heterogeneity

An important methodological issue in the present meta-analysis is the variability in the definition and assessment timing of postoperative hematoma across the included studies. Most studies defined hematoma based on ultrasound-detected hypoechoic or anechoic fluid collections greater than 1 cm in the operative region within 24–48 hours after the procedure, whereas some studies used a hematoma volume threshold, such as >10 mL, or did not provide a fully explicit diagnostic definition. This variability may reduce the comparability of outcome assessment across studies.

The timing of hematoma evaluation may also influence the observed incidence. Hematomas detected within the first 24 hours may primarily reflect acute procedural bleeding, whereas those assessed at 48 hours or later may include persistent oozing, delayed bleeding, or partially resolving collections. In addition, imaging-detected hematomas may not be equivalent to clinically significant hematomas requiring intervention, such as additional compression, aspiration, drainage, or prolonged observation. Therefore, the pooled estimates in this study should be interpreted as associations with study-defined postoperative hematoma rather than with a uniformly defined clinical endpoint.

Beyond heterogeneity in outcome definition and assessment timing, procedural variability may also have contributed to differences in hematoma occurrence across studies. To improve methodological transparency, we extracted available procedural information from the original studies and summarized it descriptively in **Supplementary Table 2**. Although all included studies involved ultrasound-guided vacuum-assisted breast lesion removal or excision using a vacuum-assisted biopsy system, the terminology and procedural details were not fully uniform. These differences included device system, needle gauge, local anesthesia or injection method, puncture route, probe position, and operator experience.

These procedural differences may affect the extent of tissue injury, the probability of vascular transection, and the effectiveness of intraoperative or postoperative hemostasis. Previous studies have suggested that differences in the gauge of vacuum-assisted excision needles may influence intraoperative bleeding and the occurrence of postoperative hematoma (30). Injection technique may modify the distance between the lesion and surrounding structures; probe position and puncture route may determine whether visible vessels or deeper tissues are injured; and operator experience may affect route planning, cutting-direction control, recognition of active bleeding, and adequacy of hemostasis. However, these procedural variables were inconsistently reported and were rarely accompanied by extractable effect estimates. Therefore, they could not be included in subgroup analyses or meta-regression. Their potential influence should be considered as a source of residual confounding and clinical heterogeneity when interpreting the pooled estimates. Future prospective studies should standardize or systematically record device type, needle gauge, injection method, puncture route, probe position, hemostatic strategy, compression protocol, and operator experience to clarify their independent contributions to hematoma risk.

### 4.4 Study limitations

Several limitations should be acknowledged in this study. (1) All included studies were observational cohort studies, and randomized controlled trials (RCTs) with stronger levels of evidence were lacking, and the strength of causal inference remains limited, the possibility of selection bias, information bias, and residual confounding could not be excluded. (2) The definition and assessment timing of postoperative hematoma were not fully standardized across the included studies. These methodological variations may introduce bias and heterogeneity, potentially affecting the robustness and generalizability of the pooled results. (3) The review protocol was not prospectively registered in PROSPERO or other public registries, and inter-reviewer agreement during study selection was not formally quantified. These factors may reduce methodological transparency and increase the possibility of selection or reporting bias. (4) Another limitation is that the meta-analysis included both adjusted and crude ORs. Although adjusted estimates were preferentially extracted, some studies only provided raw data, from which crude ORs were calculated. Because crude ORs do not account for potential confounders and the covariates included in adjusted models varied across studies, the pooled estimates may be affected by residual confounding. Future studies using standardized multivariable models or individual patient data meta-analysis are needed to confirm the independent effects of these variables. (5) The number of studies included in each individual meta-analysis was small, with fewer than ten studies available for each risk factor. This limited the statistical power of the analyses, prevented reliable quantitative assessment of publication bias, and increased the potential instability of pooled estimates. Although leave-one-out sensitivity analyses suggested that the direction of the associations was generally consistent, the findings should be interpreted as exploratory and hypothesis-generating rather than definitive. (6) Moreover, although the search period covered 1995–2025, the studies included in the quantitative synthesis were published between 2016 and 2025. Over this period, vacuum-assisted breast biopsy/excision techniques, device systems, needle gauge, hemostatic methods, compression protocols, and operator experience may have changed, potentially introducing additional clinical heterogeneity. Because these procedural factors were not consistently reported, their influence could not be quantitatively assessed.

## 5 Conclusion

In summary, this study identified five study-level factors associated with postoperative hematoma after ultrasound-guided vacuum-assisted breast lesion excision using a vacuum-assisted biopsy system: tumor numbers, a higher number of cutting passes, non-moderate tumor depth, including superficial or deep tumor, higher vascularity grade, and postoperative compression duration <48 h. The proposed T-P-B framework may help organize these factors into tumor-related, position-related, and breast- or peri-procedural management-related dimensions, thereby providing a structured approach to perioperative risk assessment and targeted hematoma prevention.

## Data Availability

The original contributions presented in the study are included in the article/**Supplementary Material**. Further inquiries can be directed to the corresponding authors.
