# Supplementary figures and images for: Study-level factors associated with hematoma after ultrasound-guided vacuum-assisted breast lesion excision: a systematic review and meta-analysis using a T-P-B framework
Source: Front Oncol. 2026 Jun 19;16:1828439. doi: 10.3389/fonc.2026.1828439 (PMC13327967; doi:10.3389/fonc.2026.1828439)

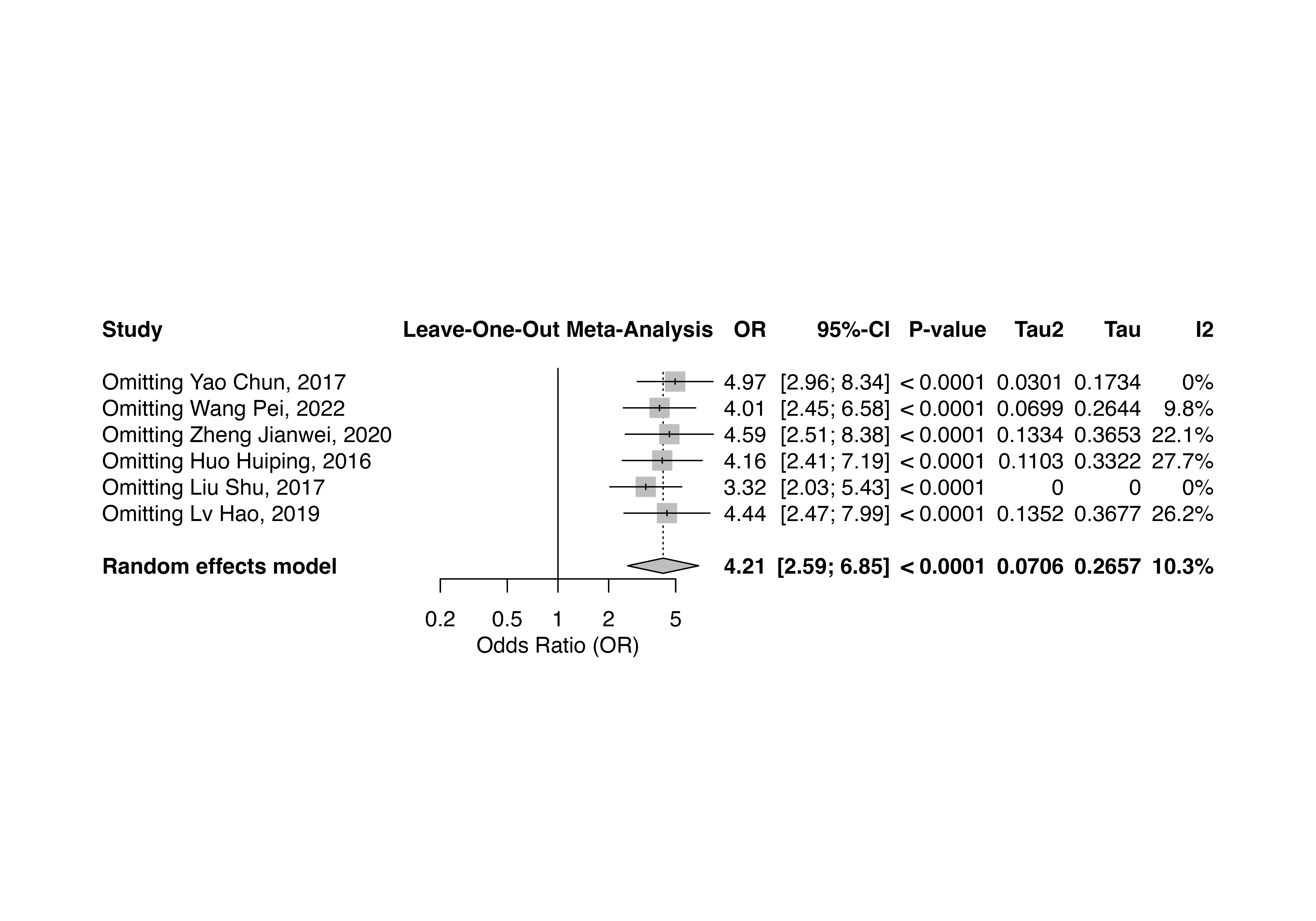

Supplement: Supplementary file 1 [file Image1.tiff]

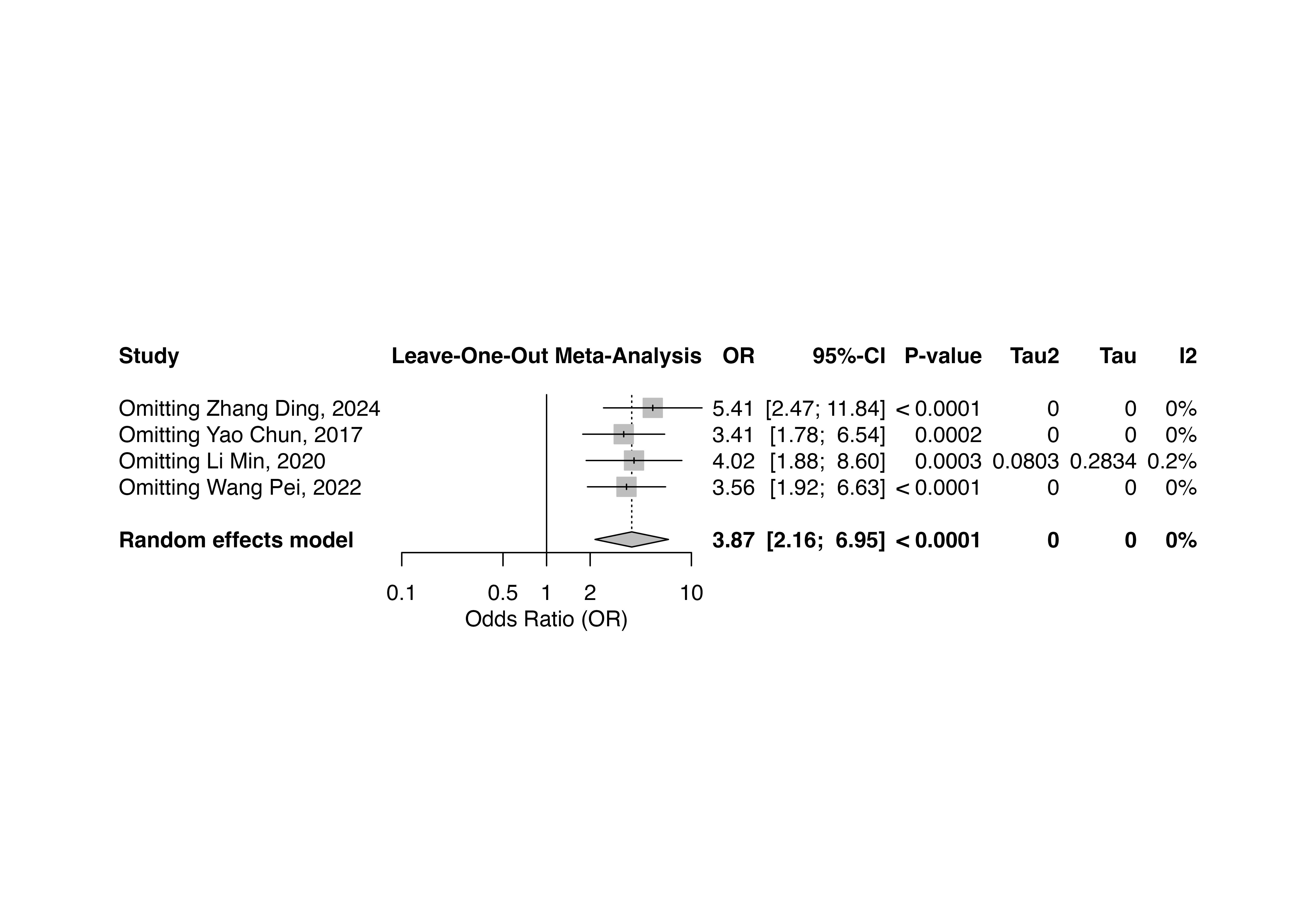

Supplement: Supplementary file 2 [file Image2.tiff]

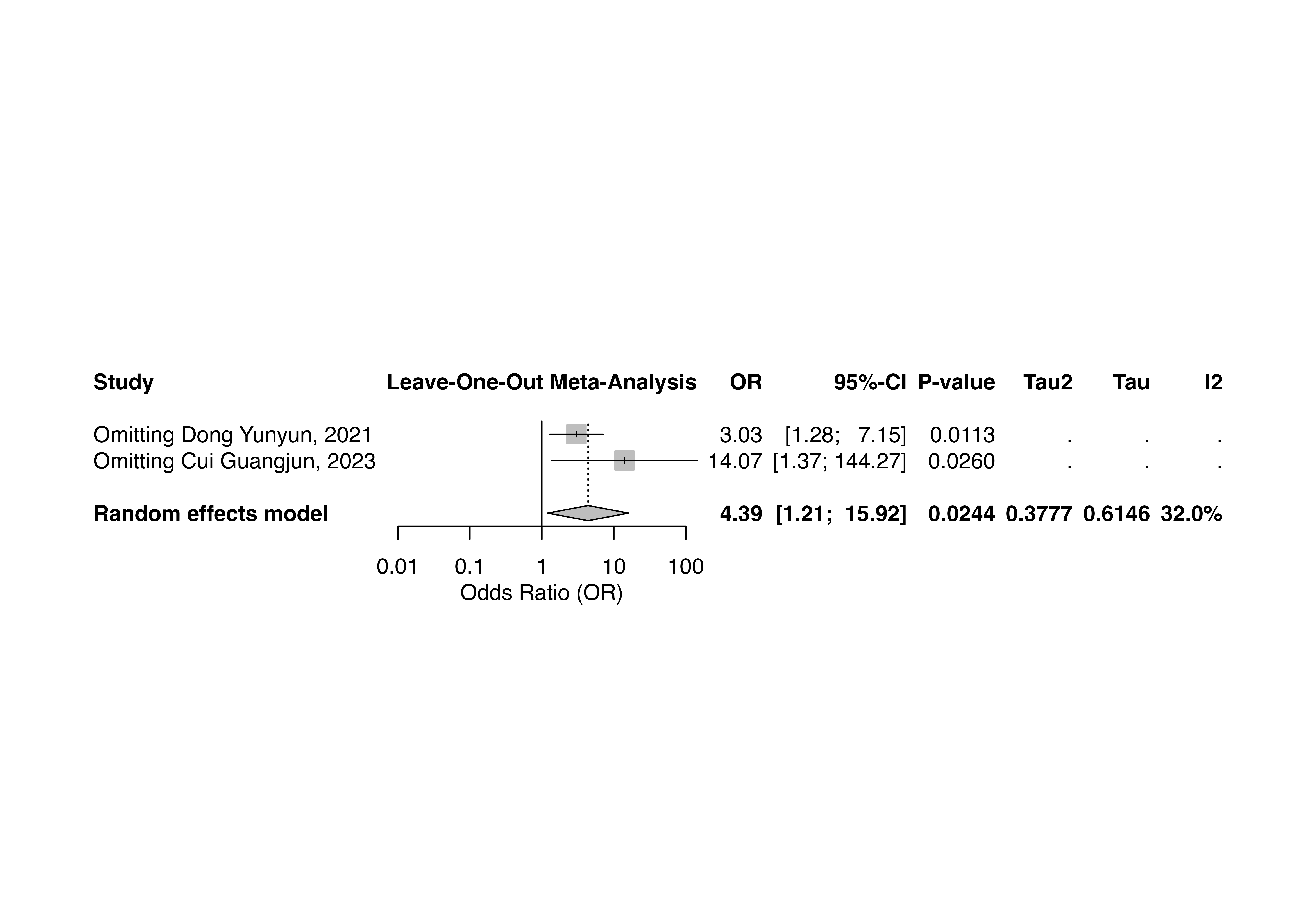

Supplement: Supplementary file 3 [file Image3.tiff]

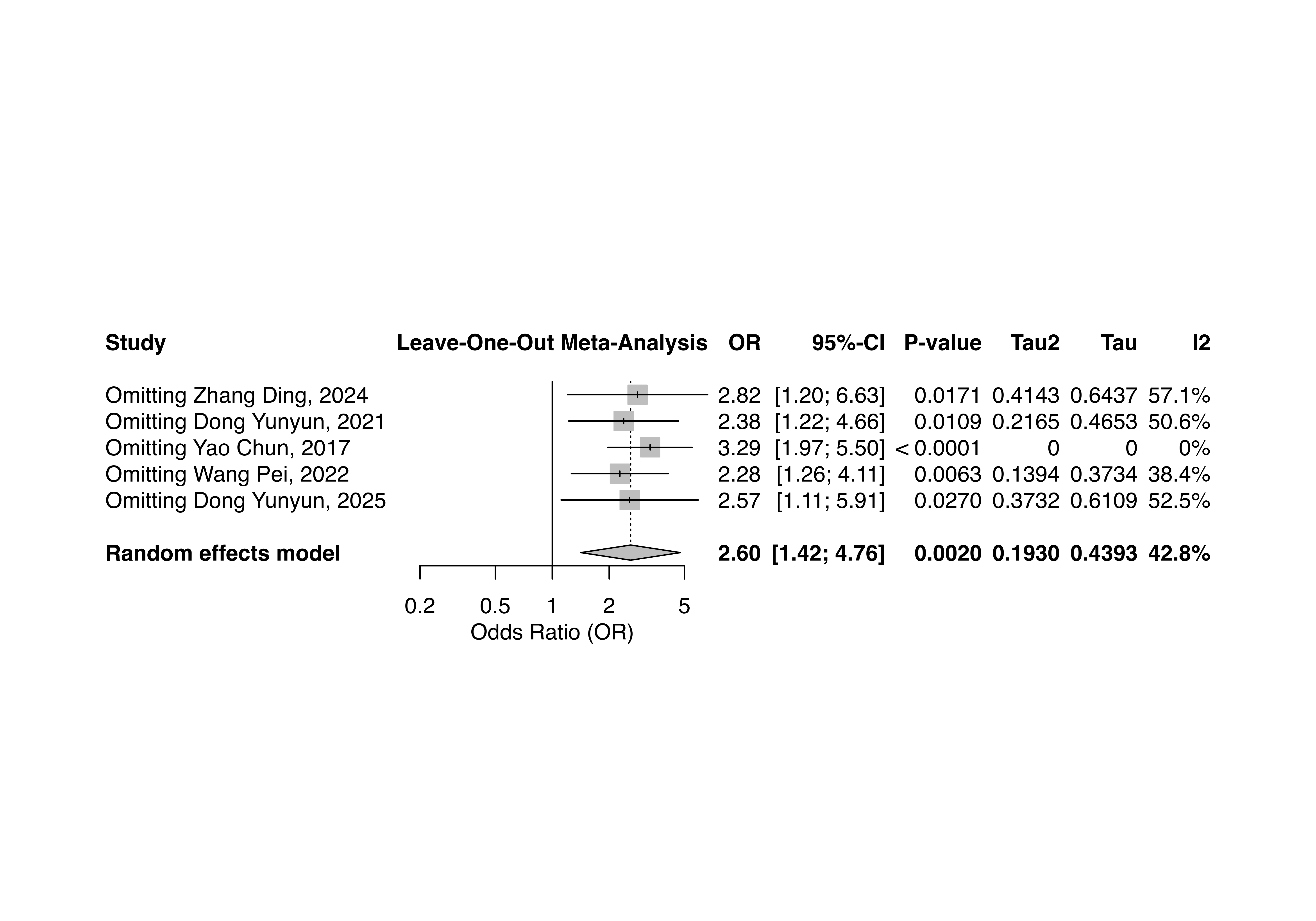

Supplement: Supplementary file 4 [file Image4.tiff]

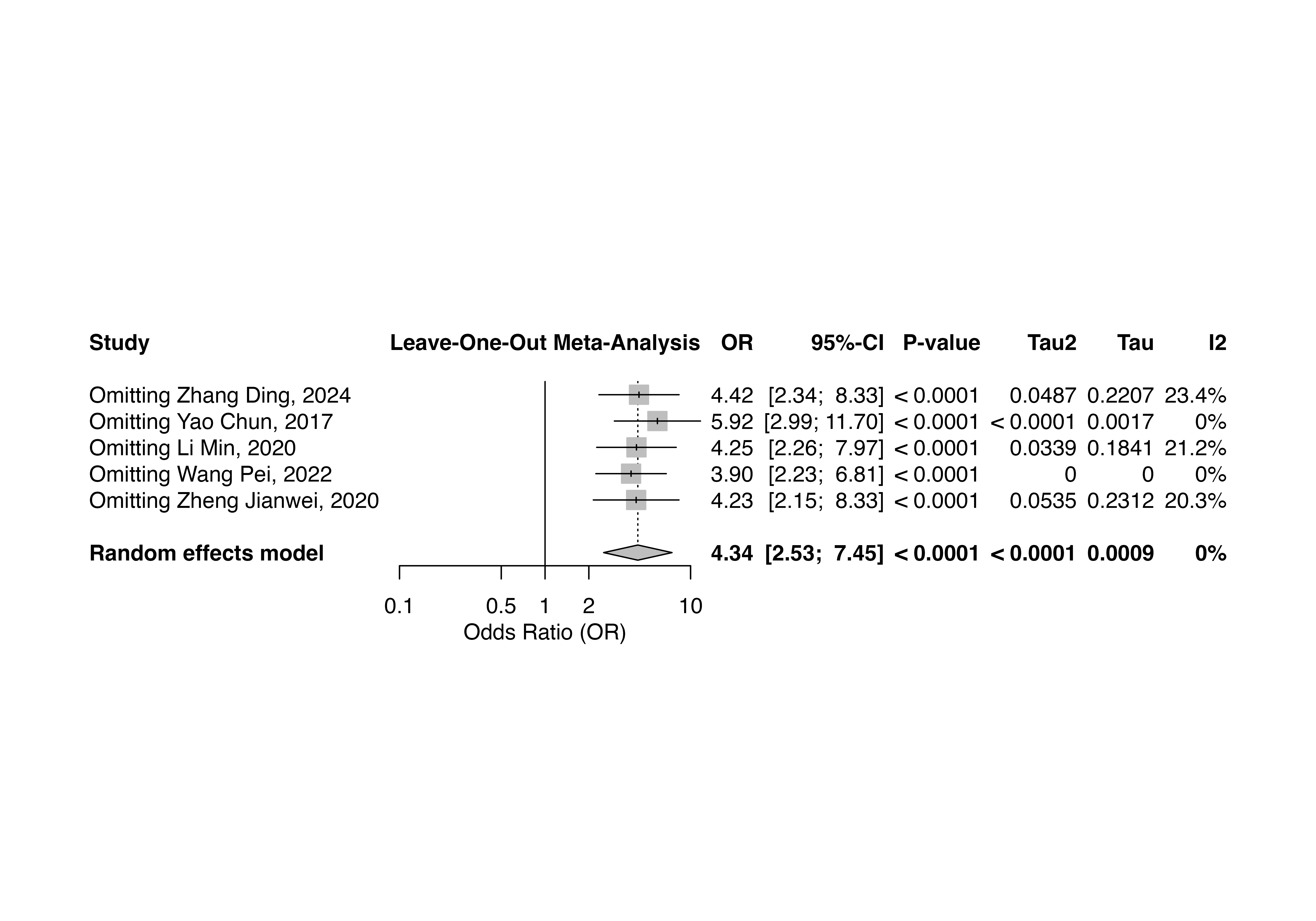

Supplement: Supplementary file 5 [file Image5.tiff]

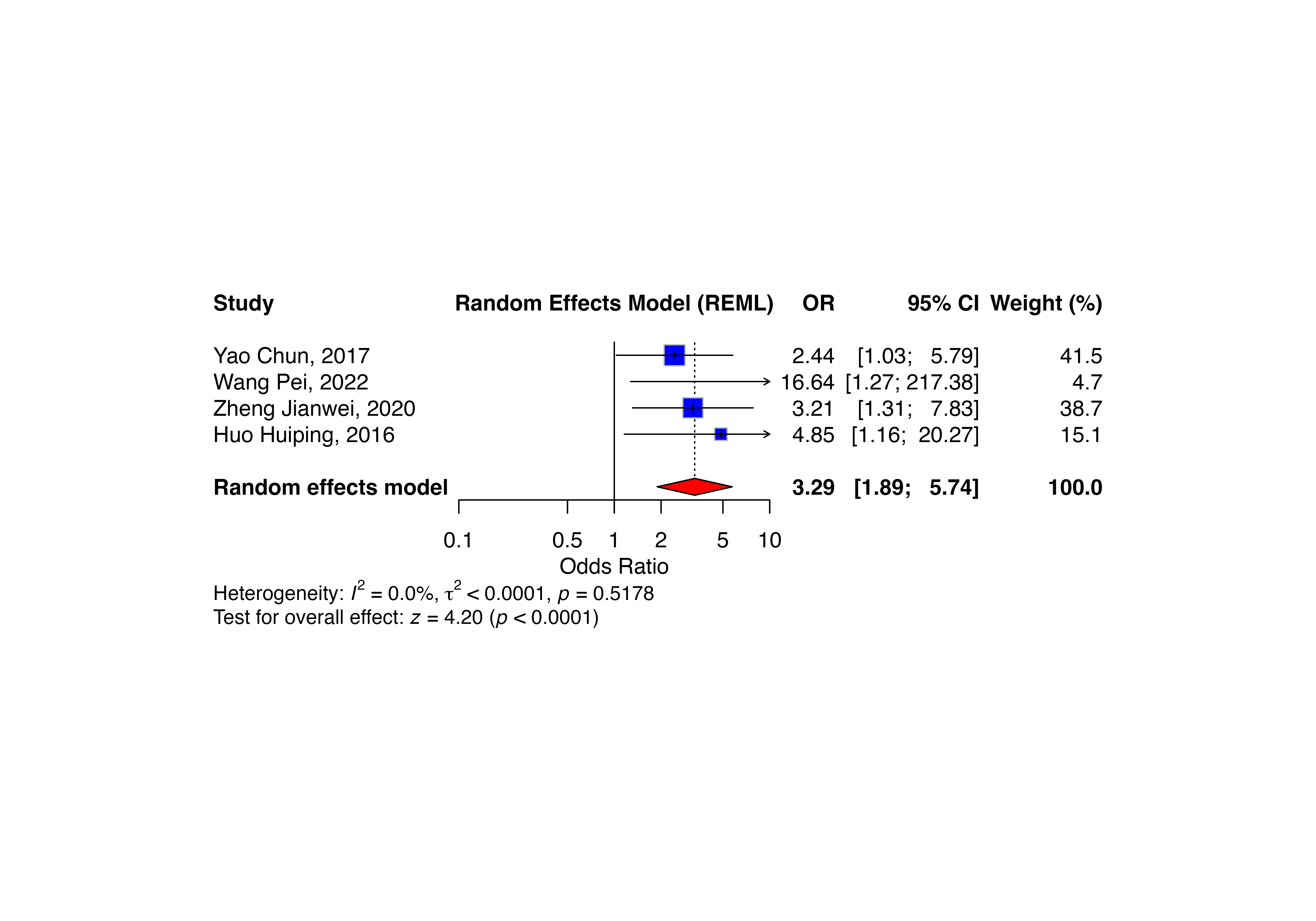

Supplement: Supplementary file 6 [file Image6.tiff]
