# Supplementary material for: Study-level factors associated with hematoma after ultrasound-guided vacuum-assisted breast lesion excision: a systematic review and meta-analysis using a T-P-B framework
Source: Front Oncol. 2026 Jun 19;16:1828439. doi: 10.3389/fonc.2026.1828439 (PMC13327967; doi:10.3389/fonc.2026.1828439)
Supplement: Supplementary file 7 [file Table1.docx]

**Supplementary Table1. Search strategies for each database**

Search period: January 1, 1995 to October 25, 2025.

| **Database** | **Search field** | **Search strategy** |
| --- | --- | --- |
| PubMed | MeSH Terms; Title/Abstract | **#1 Procedure/device terms:** ("Vacuum-Assisted Biopsy"[MeSH Terms] OR "vacuum-assisted breast biopsy"[Title/Abstract] OR "vacuum assisted breast biopsy"[Title/Abstract] OR VABB[Title/Abstract] OR Mammotome[Title/Abstract] OR EnCor[Title/Abstract])  **#2 Hematoma/bleeding terms:** ("Hematoma"[MeSH Terms] OR hematoma[Title/Abstract] OR haematoma[Title/Abstract] OR hemorrhage[Title/Abstract] OR haemorrhage[Title/Abstract] OR bleeding[Title/Abstract])  **#3 Risk/association terms:** ("Risk Factors"[MeSH Terms] OR "risk factor"[Title/Abstract] OR "risk factors"[Title/Abstract] OR predictor[Title/Abstract] OR predictors[Title/Abstract] OR prediction[Title/Abstract] OR associated[Title/Abstract] OR association[Title/Abstract] OR "influencing factor"[Title/Abstract] OR "influencing factors"[Title/Abstract])  **Final search: #1 AND #2 AND #3** |
| China National Knowledge Infrastructure (CNKI) | Subject; | **#1 Procedure/device terms:** (真空辅助乳腺活检 [vacuum-assisted breast biopsy] OR 真空辅助活检 [vacuum-assisted biopsy] OR 乳腺真空辅助活检 [breast vacuum-assisted biopsy] OR 真空辅助乳腺旋切 [vacuum-assisted breast rotary excision] OR 乳腺旋切 [breast rotary excision] OR 乳腺微创旋切 [minimally invasive breast rotary excision] OR 微创旋切 [minimally invasive rotary excision] OR 麦默通 [Mammotome] OR Mammotome OR EnCor OR VABB)  **#2 Hematoma/bleeding terms:** (血肿 [hematoma] OR 出血 [bleeding/hemorrhage] OR 术后出血 [postoperative bleeding] OR 迟发性出血 [delayed bleeding] OR 并发症 [complication])  **#3 Risk/association terms:** (危险因素 [risk factor] OR 影响因素 [influencing factor] OR 相关因素 [associated factor] OR 预测因素 [predictive factor] OR 预警因素 [warning factor] OR 因素分析 [factor analysis])  **Final search: #1 AND #2 AND #3** |
| Wanfang Data | Subject/Title; | **#1 Procedure/device terms:** (真空辅助乳腺活检 [vacuum-assisted breast biopsy] OR 乳腺真空辅助活检 [breast vacuum-assisted biopsy] OR 真空辅助活检 [vacuum-assisted biopsy] OR 真空辅助旋切 [vacuum-assisted rotary excision] OR 乳腺旋切 [breast rotary excision] OR 乳腺微创旋切 [minimally invasive breast rotary excision] OR 微创旋切 [minimally invasive rotary excision] OR 麦默通 [Mammotome] OR Mammotome OR EnCor OR VABB)  **#2 Hematoma/bleeding terms:** (血肿 [hematoma] OR 出血 [bleeding/hemorrhage] OR 术后出血 [postoperative bleeding] OR 迟发性出血 [delayed bleeding] OR 并发症 [complication])  **#3 Risk/association terms:** (危险因素 [risk factor] OR 影响因素 [influencing factor] OR 相关因素 [associated factor] OR 预测因素 [predictive factor] OR 预警因素 [warning factor] OR 因素分析 [factor analysis])  **Final search: #1 AND #2 AND #3** |
| VIP Database | Subject; | **#1 Procedure/device terms:** (真空辅助乳腺活检 [vacuum-assisted breast biopsy] OR 真空辅助活检 [vacuum-assisted biopsy] OR 真空辅助旋切 [vacuum-assisted rotary excision] OR 乳腺旋切 [breast rotary excision] OR 微创旋切 [minimally invasive rotary excision] OR 乳腺微创旋切 [minimally invasive breast rotary excision] OR 麦默通 [Mammotome] OR Mammotome OR EnCor OR VABB)  **#2 Hematoma/bleeding terms:** (血肿 [hematoma] OR 出血 [bleeding/hemorrhage] OR 术后出血 [postoperative bleeding] OR 迟发性出血 [delayed bleeding] OR 并发症 [complication])  **#3 Risk/association terms:** (危险因素 [risk factor] OR 影响因素 [influencing factor] OR 相关因素 [associated factor] OR 预测因素 [predictive factor] OR 预警因素 [warning factor])  **Final search: #1 AND #2 AND #3** |
| Elsevier ClinicalKey | All fields / Title / Abstract | **#1 Procedure/device terms:** ("vacuum-assisted breast biopsy" OR "vacuum assisted breast biopsy" OR VABB OR Mammotome OR EnCor OR "vacuum-assisted biopsy" OR "vacuum assisted biopsy")  **#2 Hematoma/bleeding terms:** (hematoma OR haematoma OR hemorrhage OR haemorrhage OR bleeding)  **#3 Risk/association terms:** ("risk factor" OR "risk factors" OR predictor OR predictors OR prediction OR associated OR association OR "influencing factor" OR "influencing factors")  **Final search: #1 AND #2 AND #3** |

**Note**: The terms listed for Chinese databases are shown with English translations in square brackets for reporting purposes. The actual Chinese terms were used in the database searches.

**Supplementary Table2. Procedural characteristics and potential procedural confounders of the included studies**

| **Study** | **Procedural terminology used in the original study** | **Vacuum-assisted device/system** | **Ultrasound system** | **Needle gauge** | **Local anesthesia/injection method** | **Excision approach/probe position** | **Operator experience** |
| --- | --- | --- | --- | --- | --- | --- | --- |
| **Dong Yunyun 2025** | Ultrasound-guided vacuum-assisted excision | EnCor (SenoRX, Irvine, CA, USA) | Hitachi Arietta 70 (Hitachi Medical, Japan) | 7G | Local anesthetic was injected into the incision site and around the nodule. | The device was advanced along the preset needle path to the lower edge of the nodule. | One operator with >10 years of experience and one operator with >3 years of experience |
| **Zhang Ding 2024** | Ultrasound-guided vacuum-assisted circumcision | Not reported | Color Doppler ultrasound diagnostic instrument with an LA523 linear-array probe; frequency set at 13 MHz | 8G | Local anesthetic was injected above, below, anterior, and posterior to the mass. | The rotary cutting probe was positioned posterior to the mass for excision. | Not reported |
| **Cui Guangjun 2023** | Ultrasound-guided vacuum-assisted minimally invasive rotary excision | Not reported | Not reported | Not reported | Not reported | Not reported | Not reported |
| **Wang Pei 2022** | EnCor minimally invasive rotary excision | Not reported | Not reported | Not reported | Lidocaine was injected to separate the mass from the surrounding tissues and create an isolation zone. | The rotary cutting probe was advanced to the base of the mass. | Not reported |
| **Dong Yunyun 2021** | Minimally invasive rotary excision of benign breast nodules | EnCor CART01 | ARIETTA 70 color Doppler ultrasound diagnostic system (Hitachi, Japan) | 7G | 1% lidocaine containing 0.1% epinephrine was injected along the preset needle tract, separating the nodule from surrounding tissues to form an isolation zone. | Cutting was initiated after reaching the basal layer of the nodule. | Not reported |
| **Zheng Jianwei 2020** | US-guided breast vacuum-assisted biopsy | 7G EnCor system (SenoRX, Irvine, CA, USA) | Siemens Acuson Sequoia 512 ultrasound system (Siemens, Erlangen, Germany) with a 15L8w linear-array probe | 7G | Local anesthesia was used. For masses adjacent to the pectoralis major muscle or just beneath the skin, lidocaine was administered between the relevant structures and the masses. | The probe was inserted into the bottom or side of the lesions. | Operator with 15 years of experience |
| **Li Min 2020** | Minimally invasive rotary excision for benign breast tumors | Not reported | Not reported | Not reported | Not reported | Not reported | Not reported |
| **Lv Hao 2019** | Mammotome minimally invasive rotary excision for benign breast masses | Mammotome vacuum-assisted breast biopsy system (Johnson & Johnson, USA) | Mindray M5 color ultrasound diagnostic system | 8G | Anesthetic was injected along the puncture tract, the fat space above the tumor, and the posterior breast space. | The rotary cutting probe was advanced beneath the mass. | Not reported |
| **Yao Chun 2017** | Ultrasound-guided rotary excision of breast masses | EnCor system (SenoRx) | Esaote MyLab 90 color Doppler ultrasound system (Italy) with an LA523 linear-array probe; frequency 13 MHz | 7G and 10G | Anesthetic was injected anterior, posterior, superior, and inferior to the mass. | The rotary cutting needle was accurately advanced posterior to the mass. | Not reported |
| **Liu Shu 2017** | Ultrasound-guided vacuum-assisted Mammotome excision | Not reported | Color ultrasound guidance with a 7-16 MHz range (SonoScape S30) | 8G | Local anesthesia was used, with anesthetic injected from the incision to the lesion along the access route. | The rotary cutting probe was placed directly beneath the mass. | At least 5 years of experience |
| **Huo Huiping 2016** | Ultrasound-guided vacuum-assisted breast excisional biopsy procedure | EnCor system (SenoRx, Aliso Viejo, CA, USA) | Siemens Acuson Sequoia 512 ultrasound system (Acuson, Mountain View, CA, USA) with a 15L8w linear-array probe | 7G | A total of 10 mL of 1% lidocaine containing epinephrine at 1:100,000 was administered into the cutaneous layer, around the lesions, and along the insertion course of the probe. | The probe was inserted into the bottom or side of the lesions, with the tubular sampling groove directed toward the lesions. | Not reported |

**Note:** Information was extracted from the original included studies when available. “Not reported” indicates that the corresponding procedural detail was not explicitly available in the original article. These procedural characteristics were summarized descriptively to clarify potential confounders and sources of clinical heterogeneity; they were not included in the quantitative synthesis because reporting was inconsistent and extractable effect estimates were generally unavailable.

**Supplementary Table 3.** NOS quality assessment of the included studies

| **Study** | **Selection** | **Comparability** | **Outcome** | **Total score** | **Quality level** |
| --- | --- | --- | --- | --- | --- |
| **Zhang Ding 2024** | 4 | 2 | 2 | 8 | High |
| **Dong Yunyun 2021** | 4 | 2 | 1 | 7 | High |
| **Liu Shu 2017** | 4 | 1 | 0 | 5 | Moderate |
| **Yao Chun 2017** | 4 | 2 | 3 | 9 | High |
| **Li Min 2020** | 4 | 2 | 3 | 9 | High |
| **Wang Pei 2022** | 4 | 2 | 3 | 9 | High |
| **Dong Yunyun 2025** | 4 | 2 | 2 | 8 | High |
| **Lv Hao 2019** | 4 | 1 | 1 | 6 | Moderate |
| **Zheng Jianwei 2020** | 4 | 1 | 3 | 8 | High |
| **Huo Huiping 2016** | 4 | 1 | 3 | 9 | High |
| **Cui Guangjun 2023** | 4 | 1 | 3 | 8 | High |

**Note:** Study quality was assessed using the Newcastle–Ottawa Scale (NOS), which evaluates observational studies across three domains: selection (0–4 points), comparability (0–2 points), and outcome assessment (0–3 points), with a total score ranging from 0 to 9.
